# Radiation-Induced Synchronous Parathyroid Carcinoma and Papillary Thyroid Carcinoma: Clinical, Morphological, and Genetic Insights

**DOI:** 10.3390/ijms26094441

**Published:** 2025-05-07

**Authors:** Gábor Iványi, Alexandros Christofi, Gábor Sipka, Tamás Zombori, Levente Kuthi, Andrea Simon, Deján Dobi, György Lázár, Zsuzsanna Valkusz, Béla Iványi

**Affiliations:** 1 Department of Internal Medicine, Albert Szent-Györgyi Medical School, University of Szeged, 6722 Szeged, Hungary; ivanyi.gabor@med.u-szeged.hu (G.I.); christofi.alexandros@med.u-szeged.hu (A.C.); valkusz.zsuzsanna@med.u-szeged.hu (Z.V.); 2 Department of Nuclear Medicine, Albert Szent-Györgyi Medical School, University of Szeged, 6720 Szeged, Hungary; sipka.gabor@med-u-szeged.hu; 3 Department of Pathology, Albert Szent-Györgyi Medical School, University of Szeged, 6725 Szeged, Hungary; zombori.tamas@med.u-szeged.hu; 4 Department of Surgical and Molecular Pathology, Tumor Pathology Center, National Institute of Oncology, 1122 Budapest, Hungary; kuthi.levente@oncol.hu (L.K.); simon.andrea@oncol.hu (A.S.); 5 Department of Pathology and Experimental Cancer Research, Semmelweis University, 1086 Budapest, Hungary; 6 HUN-REN-ONKOL-TTK-HCEMM Oncogenomics Research Group, National Institute of Oncology, 1122 Budapest, Hungary; 7 Department of Pathology, Forensic and Insurance Medicine, Semmelweis University, 1091 Budapest, Hungary; 8 Department of Surgery, Albert Szent-Györgyi Medical School, University of Szeged, 6720 Szeged, Hungary; lazar.gyorgy@med.u-szeged.hu

**Keywords:** *CDC73*, E-cadherin, hypercalcemia, next-generation sequencing, parafibromin, parathyroid carcinoma, parathyroid hormone, radiation-induced tumorigenesis, papillary thyroid carcinoma, primary hyperparathyroidism

## Abstract

The clinicopathological and molecular features of synchronous parathyroid carcinoma (PC) and thyroid carcinoma in a male patient are presented. At 11, he received mantle field radiotherapy for Hodgkin lymphoma. He had a 26-year adulthood history of recurrent nephrolithiasis treated five times with lithotripsy. At 52, he was referred to our clinic for hypercalcemia. Primary hyperparathyroidism was diagnosed (calcium: 3.46 mmol/L, parathormone: 150 pmol/L, preserved renal function, nephrolithiasis, and osteoporosis). Neck ultrasound revealed a 41 × 31 × 37 mm nodule in the left thyroid and smaller nodules in the right thyroid. Enlarged cervical lymph nodes were not observed. The large nodule was interpreted as parathyroid adenoma on 99Tc-pertechnetate scintigraphy/99Tc-MIBI scintigraphy with SPECT/CT. Total left-sided and subtotal right-sided thyroidectomy were performed. Histopathology confirmed locally invasive, low-grade PC (pT2; positive for parafibromin and E-cadherin, negative for galectin-3 and PGP9.5; wild-type expression for p53 and retinoblastoma protein; Ki-67 index 10%) and incidental papillary thyroid carcinoma (pT1b). Genetic profiling revealed no loss in *CDC73*, *MEN1*, *CCND1*, *PIK3CA*, *CDH1*, *RB1*, and *TP53* genes. Deletions in *CDKN2A*, *LATS1*, *ARID1A*, *ARID1B*, *RAD54L*, and *MUTYH* genes and monosomies in nine chromosomes were identified. The tumor mutational burden and genomic instability score were low, and the tumor was microsatellite-stable. The thyroid carcinoma exhibited a *TRIM24::BRAF* fusion. Following surgery, the parathormone and calcium levels had normalized, and the patient underwent radioiodine treatment for thyroid cancer. The follow-up of 14 months was eventless. In summary, the clinical, laboratory, and imaging features of hyperparathyroidism taken together could have suggested malignancy, then confirmed histologically. The synchronous carcinomas were most likely caused by irradiation treatment diagnosed 41 years after exposure. It seems that the radiation injury initially induced parathyroid adenoma in young adulthood, which underwent a malignant transformation around age fifty.

## 1. Introduction

Sporadic parathyroid carcinoma (PC) is a very rare endocrine malignancy and accounts for less than 1% of cases of primary hyperparathyroidism (PHPT). Sporadic PC has an indolent growth and is locally invasive, and 10–30% of patients have metastatic disease at presentation [1,2]. The factors that cause tumor initiation and cancer progression are still not known in detail. Inactivating mutations in the cell division cycle-73 gene (*CDC73*) encoding nuclear tumor suppressor protein parafibromin have been identified in up to 80% of PC samples [3,4], rendering parathyroid tissue more prone to carcinoma formation. The similarities in endocrine hypersecretion and symptoms hinder the preoperative differentiation of PC from parathyroid adenoma. In addition, making the distinction may sometimes be impossible [1]. The definite diagnosis of PC is established by a histopathological evaluation of the surgical specimen. If the invasive features are difficult to discern, an immune profile of Ki-67 proliferation index > 5%, loss of parafibromin, loss of E-cadherin, and the expression of galectin-3 and/or protein gene product 9.5 (PGP9.5) favors carcinoma over parathyroid adenoma or atypical parathyroid tumor [5,6,7]. The loss of parafibromin immunoreactivity is a pivotal step in parathyroid carcinogenesis [8]. The loss of cell-to-cell adhesion protein E-cadherin promotes invasive growth and metastatic behavior, and the expression of galectin-3 and/or PGP9.5 contributes to the proliferation and progression of cancer [5,7].

Concurrent PC and non-medullary carcinoma of the thyroid gland is an extremely rare clinical constellation. Twenty-seven such cases have been published in the English medical literature so far [9,10,11]. The clinicopathologic features of 25 cases were summarized in a review in 2022 [9]. Though synchronous cancers are commonly the consequence of field cancerization, the possible field effect etiology was not discussed in these publications, except for one that described concurrent PC and thyroid papillary microcarcinoma in a 42-year-old woman with a history of neck irradiation and chemotherapy for Hodgkin lymphoma 19 years earlier [12]. We recently evaluated and treated a middle-aged patient with features of PHPT. The preoperative diagnostic workup concluded intrathyroidal parathyroid adenoma and concurrent multinodular thyroid disease. However, the histopathological investigation of the thyroidectomy specimen diagnosed PC and synchronous incidental thyroid carcinoma. The cancers appeared to be radiation-induced, based on the medical history of the patient. The lessons learned from the diagnostic workup of PHPT in retrospect, and the morphological and molecular features of PC may merit the attention of endocrinologists, pathologists, and molecular experts of parathyroid cancer.

## 2. Case Presentation

### 2.1. Medical History, Clinical Findings

Last year, a 52-year-old Caucasian male patient was referred to our endocrinology outpatient clinic by the rheumatologist of a county hospital for non-relieving lower back pain, myalgia, hypercalcemia, hypophosphatemia, elevated alkaline phosphatase and a history of recurrent renal stones. At the age of 11, Hodgkin lymphoma was diagnosed histologically in the biopsy sample of an enlarged lymph node of the neck, classified clinically as stage III disease. He received mantle field radiation therapy (full dose: 27 Gy) and four cycles of cyclophosphamide, vincristine, procarbazine, and prednisone, followed by vinblastine in monotherapy. At the age of 26, he was admitted to the urological department of a county hospital for symptoms of renal colic. An abdominal X-ray examination identified a stone at the orifice of the left ureter, which passed without any complications. Calcium oxalate crystals were observed in the urinary sediment, and an analysis of the stone composition revealed oxalates and phosphates. The kidney stone disease recurred in the next two decades. He was treated five times with an extracorporeal shockwave lithotripsy technique in another county hospital at the age of 38, 51, and 52. We could not find any determination of the serum calcium level with an investigation of hypercalciuria in the medical documents of urological admissions.

Upon presentation, the physical examination revealed obesity (BMI: 32.84 kg/m^2^) and tenderness of the lumbar spine on percussion. A neck mass was not palpated. The family history was negative for parathyroid disease, endocrine neoplasia, and thyroid cancer. He did not complain of fatigue, abdominal pain, constipation, headaches, or depression. The initial laboratory results (Table 1) indicated severe hypercalcemia, a remarkably high parathormone level, elevated alkaline phosphatase, hypercalciuria, and preserved renal function. An X-ray examination of the thorax, vertebral column, and pelvis revealed no abnormality in the bone structure, but several small stones were observed in both kidneys. PHPT was concluded as a working diagnosis. The level of hypercalcemia was promptly lowered with saline infusions, loop diuretics, and intravenous zoledronic acid. Dual-energy X-ray absorptiometry scanning detected osteoporotic bone disease, which was most prominent in the forearms (T-score of −3.8).

### 2.2. Investigations of the Thyroid and Parathyroid Gland

**Figure 1:**
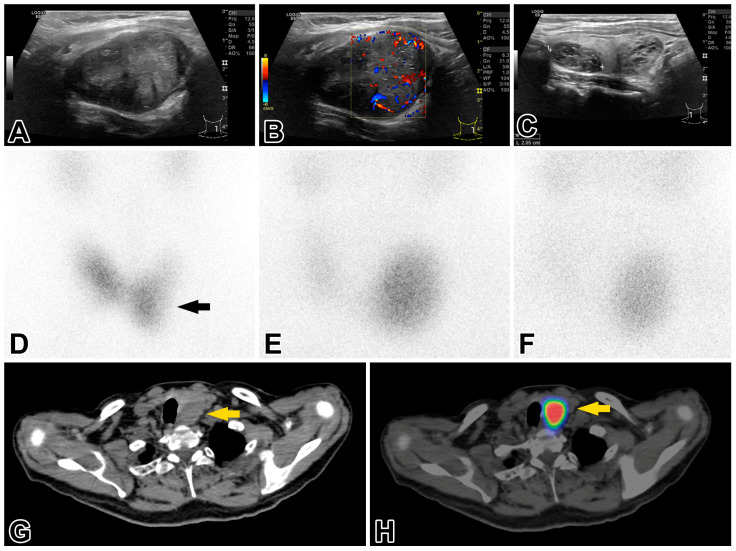
Neck ultrasound revealed (**A**) a large, hypoechoic, slightly irregularly shaped but sharply contoured, heterogeneous, solid nodule in the caudal half of the left thyroid lobe (EU-TIRADS 3); (**B**) the nodule exhibited enhanced central and peripheral vascularization; and (**C**) there were several smaller EU-TIRADS 2 nodules in the right lobe, displaying purely spongiform structures. Thyroid scintigraphy (**D**) showed a contour-deforming activity defect in the middle lower third of the left lobe, corresponding to a cold nodule (arrow). Parathyroid scintigraphy demonstrated a ^99m^Tc-MIBI-enriched nodule in the left lobe corresponding to a large parathyroid neoplasm. (**E**) The early phase (10 min) and (**F**) late phase (120 min) parathyroid SPECT/CT. (**G**) Axial slices of native (low-dose) CT images show an inhomogeneous hypodense lesion of 40 × 32 mm^2^ axial diameter in the lower half of the left thyroid lobe, not sharply demarcated from it, with a dislocation of the trachea to the right (yellow arrow). (**H**) The marked lesion displays an intensely enhanced radiopharmaceutical uptake in SPECT/CT fused images.

An initial sonographic examination of the neck, chest, and abdominal organs revealed a 41 × 31 × 43 mm EU-TIRADS 3 nodule [13] in the left thyroid lobe and four smaller EU-TIRADS 2 nodules in the right thyroid lobe. Enlarged or abnormally structured cervical lymph nodes were not detected. A fine needle aspiration biopsy (FNAB) was performed on the EU-TIRADS 3 nodule. The cytologic findings did not raise the suspicion of malignancy, and they were compatible with Hashimoto thyroiditis. The blood test for thyroid function found elevated TSH levels (7.95 mIU/L; range 0.27–4.2) and free triiodothyronine and tetraiodothyronine values close to the lower cutoff in the reference range. The level of thyroglobulin, anti-thyroglobulin antibodies, and anti-thyroid peroxidase antibodies were within normal limits. The functional data did not support the diagnosis of Hashimoto thyroiditis. The thyroid region was reexamined by an expert in ultrasonography and nuclear imaging of the thyroid (S.G). The left thyroid lobe, with dimensions 35 × 38 × 48 mm, contained a 30 × 34 × 43 mm solid nodule (Figure 1A–C) that compressed and dislocated the thyroid gland. ^99m^Tc pertechnetate thyroid scintigraphy revealed a thyroid hormone-nonproducing cold nodule in the middle lower third portion of the left thyroid lobe (Figure 1D). This nodule had been examined previously by FNAB. ^99m^Tc-MIBI subtraction parathyroid scintigraphy visualized an area of increased radioactive uptake at the lower third of the left thyroid pole in the 10 and 120 min delayed images (Figure 1E,F). Subsequent single photon emission computed tomography (SPECT/CT) of the neck and skull demonstrated a well-circumscribed, inhomogeneous rounded lesion in the enlarged left thyroid lobe that had dislocated the trachea (Figure 1G,H). Based on these findings, a preoperative diagnosis of a large intrathyroidal parathyroid adenoma and concomitant multinodular thyroid disease was established. A left-sided total and right-sided subtotal thyroidectomy was performed. The postoperative period was eventless.

### 2.3. Pathological Investigations

The cytologic smears submitted were moderately cellular and displayed numerous cohesive three-dimensional groups with oxyphil cells occasionally in the background of colloid material, many lymphocytes, and fewer macrophages. Large numbers of dissociated cells, pleomorphic cells in loosely cohesive groupings, and small nucleoli were not seen. The changes were consistent with benign thyroid disease like Hashimoto thyroiditis.

**Figure 2:**
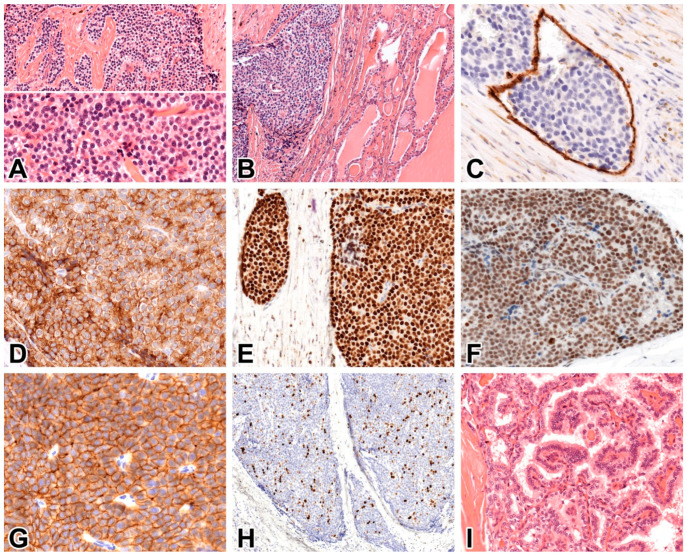
The morphological features of PC and papillary thyroid carcinoma are shown. (**A**–**H**) depict PC, and I shows thyroid carcinoma. (**A**) The solid nests and cords of tumor cells are separated by fibrous trabeculae (top). The tumor cells have round monomorphic nuclei with minimal cytologic atypia (bottom). Hematoxylin and eosin (HE); ×20 and ×40, respectively. (**B**) The carcinoma had infiltrated the thyroid follicles. HE, ×10. (**C**). A tumor thrombus in the small vein of pseudocapsule. The layer of endothelial cells is CD34-positive, ×40. (**D**) The cytoplasm of tumor cells is diffusely positive for parathormone, ×40. (**E**) The nuclei of tumor cells are diffusely positive for GATA3. The smaller nest had infiltrated the pseudocapsule, ×40. (**F**). The diffuse nuclear expression of parafibromin. The photo shows an invasive nest in the pseudocapsule, ×20. (**G**) Diffuse membranous positivity of E-cadherin in tumor cells, ×40. (**H**) Nuclear Ki-67 positivity in approximately 10% of tumor cells at hotspots, ×20. (**I**) Papillary thyroid carcinoma at medium-power micrograph. HE, ×20.

The grossing of a surgical specimen from the left side revealed a 45 × 20 × 14 mm, firm grayish white, relatively circumscribed tumorous lesion. The lesion did not have an unambiguous capsule, and it was not surrounded by normal thyroid tissue along the inked resection line. Histologically, the lesion was mostly demarcated from the thyroid follicles by a delicate connective tissue rim. The tumor cells resembled chief cells of the parathyroid gland, and they grew in solid nests or cords intervened with fibrous trabeculae focally (Figure 2A, top). The tumor cells had round monomorphic nuclei and indistinct nucleoli (Figure 2A, bottom), and there were two typical mitoses per 10 mm^2^. Prominent nucleoli, necrosis, or tumor-infiltrating lymphocytes were not observed. The infiltration of thyroid follicles in one visual field (Figure 2B) and tumor thrombi in a few CD34-positive small veins in the pseudo-capsule (Figure 2C) led us to diagnose the lesion as PC. The circumferential resection margin appeared to be positive at several microscopic sites (postoperative tumor stage: pT2, R1, V1, L0, and Pn0). The immunohistochemical profile of PC was investigated by stains for parathyroid cell marker parathormone, chromogranin A, and GATA3, as well as parafibromin, E-cadherin, galectin-3, PGP9.5, p53, retinoblastoma protein (pRb), and Ki-67 [5,7,14]. The antibodies were commercially available, and the tissue sections were stained with an immunohistochemical automated slide stainer. All the tumor cells exhibited intense positivity for parathormone (Figure 2D), chromogranin A, GATA3 (Figure 2E), parafibromin (Figure 2F), and E-cadherin (Figure 2G) in the corresponding expression sites. The Ki-67 proliferation index at hotspots was 10% (Figure 2H), p53 and pRb displayed a wild-type staining pattern, and the stainings for galectin-3 and PGP9.5 were negative.

As regards the right thyroid lobe, the grossing detected a 20 × 15 × 14 mm firm, partly cystic grayish-white nodule, which displayed histologically true papillae with a central vascular core, nuclear features of papillary carcinoma (Figure 2I), and psammoma bodies. A diagnosis of the classic variant of papillary thyroid carcinoma was established (postoperative stage: pT1b, R0, V0, L0, Pn0). Far from the carcinoma, there was a benign thyroid nodule in the thyroid parenchyma.

### 2.4. Genomic Profiling of Parathyroid Carcinoma and Papillary Thyroid Carcinoma

Formalin-fixed, paraffin-embedded tumor tissue samples were used. For next-generation sequencing (NGS), libraries were prepared using the Ion Chef™ System with Ion 540™ Chips (Thermo Fisher Scientific, Waltham, MA, USA), following the manufacturer’s instructions. DNA and RNA inputs were approximately 4 ng and 5.7 ng, respectively. Sequencing was performed with an Ion S5™ Plus Sequencer (Thermo Fisher Scientific, Waltham, MA, USA). The Oncomine™ Comprehensive Assay Plus system was used to examine the parathyroid carcinoma, and the Oncomine™ Precision Assay was applied to study the papillary thyroid carcinoma. Data analysis was conducted using Ion Reporter™ Software (v. 5.18) (Thermo Fisher Scientific, Waltham, MA, USA).

The NGS analysis of parathyroid carcinoma did not identify any pathogenic mutations or gene fusions in the *CDC73*, *MEN1*, *CCND1*, *PIK3CA*, *CDH1*, *TP53*, and *RB1* genes. However, it found deletions in the *CDKN2A*, *ARID1A*, *ARID1B*, *RAD54L*, *MUTYH*, and *LATS1* genes, along with a single nucleotide variant in CDKN2C (c.64_65insA), classified as a variant of unknown significance in existing databases. Furthermore, monosomies of chromosomes 2, 3, 8, 9, 10, 12, 13, 20, and 22 were detected, along with gains in 1q and 7p and losses in 5p and 11p. The tumor mutational burden was low (1.9 mut/Mb), and the genomic instability score was also low (HRD score 5). The *MLH1*, *MSH2*, *MSH6*, and *PMS2* mismatch repair genes had not mutated, and the tumor was microsatellite-stable. Genomic profiling of the papillary thyroid carcinoma identified a TRIM24::BRAF fusion with a read count of 2221. No other fusions or genetic alterations were detected, including the *EGFR*, *ALK*, *ROS1*, *RET*, *KRAS*, *PIK3CA*, and *ERBB2* genes.

### 2.5. Postoperative Treatment and Follow-Up

After the pathology report was validated, the multidisciplinary onco-team decided that the patient should receive radioactive iodine ablation therapy (3700 MBq), carried out three months after the thyroidectomy. A follow-up iodine full-body SPECT/CT scan found no remaining iodine-dense residue. The patient has been receiving alfacalcidol and cholecalciferol vitamin D supplementation, along with calcium supplementation ever since the operation. The patient has been complaint-free since the operation. Follow-up blood test results were normal for the serum parathormone level and almost normal for the serum calcium level, so the alfacalcidol and calcium supplementation doses were lowered. Thyroglobulin levels became immeasurably low after radioiodine therapy. The patient is regularly followed in our endocrine outpatient clinic, the last visit being 14 months after the operation. After 12 months, the calcium supplementation was suspended.

## 3. Discussion

Based on the pathological diagnosis of the thyroidectomy samples, three lessons can be drawn from the clinical history and diagnostic workup of PHPT. The patient experienced his first renal colic episode at the age of 26. The passed stone was radiodense on X-ray scans and contained oxalates and phosphates, suggesting a calcium-containing composition. If we suppose that this renal colic was the first clinical manifestation of PHPT, the endocrine abnormality remained undiagnosed for 26 years because the serum calcium level and hypercalciuria had not been checked during the urological admissions. Best practice guidelines from the U.S., U.K., Scandinavia, etc. recommend routine screening for hypercalcemia in nephrolithiasis patients [15,16,17]. However, adherence to national guidelines may not be strict in clinical practice [18]. In Hungary, no guideline on the metabolic evaluation of patients with kidney stones exists, and urologists decide on an individual basis whether recurrent stone formers should undergo serum calcium level assessment or not. The endocrine etiology of kidney stone formation was ultimately considered by a rheumatologist when the patient was 52 years old.

A screening neck ultrasound examination revealed an EU-TIRADS 3 nodule in the left thyroid lobe. FNAB smears from this nodule were misinterpreted as indicative of Hashimoto thyroiditis, as the working diagnosis of PHPT had not been established at that time. The cytologist did not know that the sample might have aspirated from a parathyroid nodule, which may display overlapping features with thyroid neoplasms, adenomatous thyroid nodules, or lymphocytic thyroiditis [19]. The misdiagnosis of parathyroid adenoma as a thyroid lesion via FNAB has been previously documented [20,21,22]. Current recommendations advise against FNAB for suspected parathyroid nodules because it is hard to distinguish parathyroid adenoma from PC, and there are concerns about the needle tract seeding of cancer cells [1,3]. In our case, the FNAB misinterpretation did not affect clinical management, as Hashimoto thyroiditis was ruled out by thyroid function tests.

Preoperative SPECT/CT imaging revealed a well-circumscribed, solitary lesion located in the lower half of the left thyroid lobe, with no lymph node metastases, supporting the diagnosis of intrathyroidal parathyroid adenoma. This variant especially challenges preoperative imaging evaluations [19,23]. In retrospect, certain sonographic features, such as a slightly irregular shape, hypoechoic and heterogeneous texture, and a size exceeding 3 cm, did not entirely support the diagnosis of adenoma. The combination of a parathormone level at least four times higher than normal (in our patient, it was 150 pmol/L instead of 28 pmol/L), a serum calcium level of ≥3.5 mmol/L, the simultaneous presence of nephrolithiasis and osteoporosis, and a parathyroid lesion exceeding 3 cm should have suggested that PC was the likely cause [1,2,3,4]. In a review of 25 cases with synchronous carcinomas of the parathyroid and thyroid gland, only five had “suspected PC” as a stated justification for surgery, despite 83% of the patients displaying parathormone levels 5–10-fold the normal value [9]. If PC is suspected, en bloc resection of the parathyroid gland along with the ipsilateral thyroid lobe and adjacent structures is justified, in contrast to the standard minimally invasive parathyroidectomy for solitary adenomas. Clinically relevant thyroid disease necessitates subtotal thyroidectomy contralaterally. In the review of 25 synchronous cases of PC and thyroid carcinoma, the latter was an incidental finding in 84% of cases, and the vast majority turned out to be papillary thyroid carcinoma [9]. In our patient, the papillary thyroid carcinoma found incidentally was 2 cm in the greatest dimension (pT1b), and the finding necessitated the radioiodine ablation of the remnant right thyroid lobe.

Four arguments supported radiation-induced tumorigenesis in our patient. First, there is a well-documented association between radiation exposure to the neck and an increased risk of developing parathyroid adenoma [24,25], parathyroid carcinoma [26], or thyroid cancer [27]. Second, the synchronous carcinomas of two organs had occurred within a previously irradiated area that induced field cancerization. Third, there was a 41-year latency period between radiation exposure and the diagnosis of synchronous cancers. Lastly, there was no evidence of a genetic predisposition, as the family history of thyroid and parathyroid disease was negative. It should also be mentioned that combining chemotherapy with radiotherapy in childhood Hodgkin lymphoma increases the risk of secondary malignancies by 4.4 times compared to 2.8 times with radiation alone [28]. Nowadays, the treatment of childhood Hodgkin lymphoma or childhood acute lymphoid leukemias still requires external beam radiotherapy, and therefore, the risk of developing radio-induced cancers decades later should be considered in survivors of childhood malignancy.

Radiation exposure induces DNA damage in tissue stem cells [29], leading to single-base damage, single-strand breaks, double-strand breaks, and DNA–protein cross-links. The repair of such damage is complex and involves multiple pathways, including base excision repair for single-base damage and single-strand breaks, nucleotide excision repair for cross-links, and homologous recombination for double-strand breaks [30]. Errors in these pathways may result in deletions, duplications, inversions, translocations, or even whole chromosomal aneuploidy [31,32].

Although the molecular signature of radiation-induced PC remains unclear, the genetic profiling of our case sheds new light on the topic. Sporadic parathyroid adenomas frequently exhibit somatic biallelic inactivation of the *MEN1* tumor suppressor gene [33,34]. Genetic alterations in cyclin-dependent kinase inhibitor genes (*CDKN1A*, *CDKN1B*, *CDKN2B*, and *CDKN2C*) appear to be infrequent [35,36]. Radiation-induced adenomas exhibit multiple losses and gains, with a relatively frequent loss of 1p and a gain of 19p [33]. Sporadic PCs commonly harbor four driver mutations, either separately or in combination. These are the biallelic inactivation of the *CDC73* gene, the amplification of the *CCND1* gene leading to cyclin D1 overexpression, alterations in the PI3K/AKT/mTOR pathway, and inactivating mutations in the tumor suppressor gene *PRUNE2* [37,38,39,40]. Loss of parafibromin due to *CDC73* mutations results in altered gene expression, cell cycle activation, and increased proliferation [8]. *CDC73*-mutant PCs exhibit a higher tumor mutational burden, genomic instability, and metastatic potential [38,40]. Germline screening is recommended for *CDC73*-mutant tumors, as in younger individuals, PC may have a syndromic background [41]. In contrast, *CDC73* wild-type PCs lack these specific features [38].

The genetic profiling in our case did not reveal any *CDC73* mutations or loss of heterozygosity, and parafibromin was diffusely expressed immunohistochemically in the nuclei of tumor cells. Furthermore, no mutations were found in the *CCND1* or *PIK3CA* genes. The *CDH1*, *TP53*, and *RB* genes were likewise not mutated, and the corresponding immunostainings exhibited normal membranous expression or wild-type patterns. *CDH1* regulates cell–cell adhesion and inhibits cell proliferation and invasion, *TP53* prevents the propagation of genetically defective cells and maintains genomic stability, and *RB* is a negative regulator of cell-cycle progression. Their biallelic loss favors a more aggressive clinical course [40].

While conventional cancer driver genes of sporadic PC were not involved in our case, deletions were identified in the tumor suppressor genes *CDKN2A*, *LATS1*, *ARID1A*, and *ARID1B*. *CDKN2A* encodes p16^INK4a^ and regulates the G1/S checkpoint. It is frequently altered in a range of cancers, including two previously reported PC cases [42]. The *LATS1* gene is a regulator of organ size and tissue regeneration. Pathogenic mutations in this gene were reported in malignant mesothelioma [43]. Deletions of *ARID1A* and *ARID1B* drive hyperproliferation and dedifferentiation in several human cancers [44]. Furthermore, deletions were identified in the homologous recombination repair *RAD54L* gene and the base excision repair *MUTYH* gene. *RAD54L* defects have also been implicated in promoting the development and progression of various cancer types [45]. *MUTYH* mutations have been linked to the *MUTYH*-associated colorectal polyposis syndrome [46]. A pathogenic variant of *MUTYH* was recently observed in one of four sporadic PC cases [40]. The mutational status of *PRUNE2* remained unknown because the Oncomine™ Comprehensive Assay Plus simply did not analyze this gene.

The third molecular feature was the presence of monosomies involving nine chromosomes. Defects in DNA repair may generate whole-chromosome aneuploidy, as incorrectly repaired double-stranded DNA breaks may block mitosis, chromosome segregation, and cytokinesis. In our case, the homologous recombination pathway for double-strand DNA repair was defective, as the *RAD54L* gene was deleted. Although aneuploid cells do not proliferate as efficiently as their diploid counterparts [47], chromosome loss may initiate carcinogenesis due to haploinsufficiency of tumor suppressor genes [32]. The tumor mutational burden and the genomic instability score were low, and the tumor was microsatellite-stable. The identified molecular features appear to correlate with the relatively “friendly-looking” histopathological and immunohistochemical characteristics of PC.

The PC lacked features of cytologic atypia in smears. Histologically, it appeared as a low-grade tumor, with minimal cytologic atypia and low mitotic activity, invasion of vascular channels, and extension to thyroid follicles. Except for the Ki-67 proliferation index being 10%, the immune profile (positive for parafibromin and E-cadherin, negative for galectin-3 and PGP9.5, and wild-type expression of p53 and pRb) was not suggestive of PC. In a recent study of 44 PCs [7], parafibromin loss was observed in 54.5%, Ki-67 index >5% in 45.5%, E-cadherin loss in 47.7%, positive galectin-3 staining in 61.4%, and positive PGP9.5 staining in 38.6% of cases, and the loss of parafibromin and E-cadherin, along with a Ki-67 index of >5%, favored PC. Regrettably, biomarker expressions in localized PC vs. metastatic PCs were not analyzed separately, and different conclusions probably would have been drawn if the two groups had not been lumped together. In another study on parafibromin expression in 53 cases of PC [14], the loss of parafibromin was observed in all carcinomas with metastasis (17/17) and 14 of the 36 carcinomas with only local infiltration. The parafibromin staining was positive in all the adenomas (*n* = 53). The preserved parafibromin expression in 61% of locally invasive PC cases in that study and in our locally invasive case indicates that early PCs can frequently be parafibromin-positive, and the differentiation of parafibromin-expressing PC from parathyroid adenoma still relies on the meticulous histological search for invasiveness.

The most frequent driver mutation in both radiation-induced and sporadic papillary thyroid carcinoma comes from the genome rearrangements between the *RET* gene and the *PTC* genes (*CCDC6* and *NCOA4*), located close to each other on chromosome 10. The *RET* gene encodes a receptor tyrosine kinase located at the cell membrane. Constitutively active *RET/PTC* fusion protein is generated via the intrachromosomal inversion of *RET* and *PTC* genes, and the protein stimulates the mitogen-activated protein kinase (MAPK) pathway, which gives rise to increased cell proliferation and resistance to apoptosis [31]. The activation of the MAPK pathway via T1799A point mutation in the *BRAF* gene, which gives rise to the BRAF^V600E^ protein, appeared to be relatively infrequent in radio-induced thyroid cancers [48]. In our case, the genomic profiling of papillary thyroid carcinoma led us to identify a *TRIM24::BRAF* fusion. The close position of the two genes at chromosome 7q33-34 was the basis of intrachromosomal rearrangement that brought about kinase fusion-positive thyroid carcinoma [49]. In a review of 25 synchronous cases of PC and non-medullary thyroid carcinoma, thyroid cancer was incidental in 84% of cases, and 88% of thyroid cancers proved to be papillary thyroid carcinomas [9]. The postoperative tumor stage was described in 21 cases. The majority (57%) were not more than 1 cm (pT1a), 14% ranged between 1 and 2 cm (pT1b), and 24% between 2 and 4 cm (pT2). One case had grown beyond the thyroid (pT4).

## 4. Conclusions

Although nephrolithiasis is the target organ manifestation of PHPT, not all urologists have the necessary diagnostic experience in such cases. Therefore, campaigns should be organized to encourage the routine investigation of the serum calcium level and hypercalciuria for every patient with recurrent kidney stones, with referral of positive cases to the endocrinology department. Further, solitary hyperfunctioning parathyroid lesions may sometimes be PCs, and diagnostic vigilance is needed to ‘catch’ such cases. If the lesion exceeds 3 cm and the parathormone level is four times or more above normal, then malignancy should be suspected.

The synchronous carcinomas in our patient appeared to be the late consequence of irradiation treatment of the neck for childhood Hodgkin lymphoma. The decades-long history of recurrent kidney stone disease as a manifestation of PHPT and the low-grade appearance of PC with an adenomatous immunohistochemical profile together suggest that the radiation injury initially induced parathyroid adenoma in young adulthood, which underwent a malignant transformation around age fifty. The molecular signature of PC lacked mutations in conventional cancer driver genes of sporadic PC but displayed deletions in certain tumor suppressor genes and DNA repair genes and monosomies of nine chromosomes. The thyroid carcinoma harbored a *TRIM::BRAF* fusion. Our case is the first to provide a comprehensive immunohistochemical and genetic profile of a PC that was probably induced by radiation therapy.

## Figures and Tables

**Table 1:** Serum laboratory values on the first evaluation.

	Measured Value	Normal Value Range
Sodium (mmol/L)	136	136–145
Potassium (mmol/L)	4.8	3.5–5.1
Calcium (mmol/L)	3.48	2.20–2.55
Albumin-adjusted calcium (mmol/L)	3.46	2.20–2.55
Magnesium (mmol/L)	0.73	0.7–1.05
Phosphorus (mmol/L)	0.51	0.87–1.45
Alkaline phosphatase (U/L)	463	35–104
Carbamide (mmol/L)	6.4	2.1–7.1
Creatinine (µmol/L)	96	53–97
eGFR-EPI (ml/min/1.73 m^2^)	77	90<
Parathormone (pmol/L)	150	1.6–6.9
Urinary calcium excretion (mmol/L)	423	1.25–3.75

## Data Availability

Sequence data sets are unavailable due to privacy and ethical restrictions.
